# Sex influences susceptibility to methamphetamine cardiomyopathy in mice

**DOI:** 10.14814/phy2.14036

**Published:** 2019-03-19

**Authors:** Marie C. Marcinko, April L. Darrow, Aaron J. Tuia, Ralph V. Shohet

**Affiliations:** ^1^ Department of Medicine University of Hawaii John A. Burns School of Medicine Honolulu Hawaii

**Keywords:** Cardiomyopathy, gender, methamphetamine, transcription

## Abstract

In this study, we created a mouse model of methamphetamine cardiomyopathy that reproduces the chronic, progressive dosing commonly encountered in addicted subjects. We gradually increased the quantity of methamphetamine given to C57Bl/6 mice from 5 to 40 mg/kg over 2 or 5 months during two study periods. At the fifth month, heart weight was increased, echocardiograms showed a dilated cardiomyopathy and survival was lower in males, with less effect in females. Interestingly, these findings correspond to previous observations in human patients, suggesting greater male susceptibility to the effects of methamphetamine on the heart. Transcriptional analysis showed changes in genes dysregulated in previous methamphetamine neurological studies as well as many that likely play a role in cardiac response to this toxic stress. We expect that a deeper understanding of the molecular biology of methamphetamine exposure in the heart will provide insights into the mechanism of cardiomyopathy in addicts and potential routes to more effective treatment.

## Introduction

Methamphetamine (Meth) is a highly toxic and addictive drug that stimulates the central nervous system by triggering the release and inhibiting the reuptake of norepinephrine and dopamine, leading to a variety of excitatory neurologic responses. The dopaminergic stimulation may contribute to the sense of euphoria and the adrenergic effects to the heightened alertness that are hallmarks of the drug. The physiological and pharmacological study of methamphetamine has focused on the neuropsychological effects of the drug. However, the adrenergic stimulation also affects cardiovascular tissues and hemodynamic effects are readily seen at the doses commonly used in humans. Cardiovascular toxicity has been recognized for over 30 years and high catecholamine levels are known to be cardiotoxic, causing narrowing and spasm of the blood vessels, tachycardia, hypertension, stroke, and cardiomyocyte death. These findings are well‐recognized as acute effects of methamphetamine intoxication. In addition, chronic abuse at high levels of the drug addiction can also lead to a dilated cardiomyopathy, often of remarkable severity.

Cardiomyopathy is a common cause of death among long‐term addicts and appears to be on the rise as the average duration of drug use increases (Herbeck et al. 2015). Presentation is gradual and insidious with typical symptoms of biventricular heart failure. Long‐term studies of chronic methamphetamine abuse have suggested a strong relationship between duration of drug use and cardiomyopathy (Kaye et al. 2007). It appears that these individuals are also at risk for accelerated development of coronary artery disease. Ventricular dysfunction rarely improves upon discontinuation of the drug or the addition of medical therapies (Jacobs 1989; Lopez et al. 2009).

How this drug causes cardiomyopathy is not known, but more progress has been made in understanding the mechanism of neurotoxicity in chronic methamphetamine abuse (Tata and Yamamoto 2007). Such use produces long‐lasting deficits in dopamine and serotonin nerve terminals with signs of axonal degeneration (Yamamoto et al. 2010). High doses of methamphetamine produce an increase in extracellular and intracellular dopamine that can rapidly auto‐oxidize to form potentially toxic substances (Yamamoto et al. 2010) (Krasnova and Cadet 2009). It is this oxidation, as well as elevated levels of glutamate and mitochondrial dysfunction, that is thought to damage dopamine terminals and thus neurons (Tata and Yamamoto 2007; Krasnova and Cadet 2009).

Human studies demonstrate that males have greater dopamine release than women in response to methamphetamine, and may exhibit an increase in the degree of toxicity, suggested by a higher incidence of emergency department‐related deaths involving meth (Dluzen and Liu 2008). In rodent studies, male mice also display greater meth‐induced neurotoxicity than females (Wagner et al. 1993; Bourque et al. 2011).

These differences may result from estrogen. It has been reported that exogenous estrogen preserves striatal dopamine concentrations in gonadectomized female mice following meth administration (Dluzen and McDermott 2000). A similar neuroprotective response has been seen in estrogen‐treated intact female mice (Dluzen and McDermott 2002). This neuroprotective effect occurs quickly and may be related to estrogen inhibiting meth uptake in the brain (Dluzen and McDermott 2006). Conversely, no neuroprotection by estrogen against meth was evident in either intact or gonadectomized male mice, suggesting that testosterone may increase neurotoxicity (Dluzen and McDermott 2006).

Compared to the brain, there has been little investigation of methamphetamine toxicity in the heart. It has been recognized that high doses of meth can cause vasoconstriction, myocardial infarction and fibrosis (Yu et al. 2002). In addition, meth directly depresses contractile function, intracellular Ca^+2^ handling and adrenergic response in isolated cardiomyocytes (Turdi et al. 2009). These early studies do not explore the long‐term effects of meth exposure. Since human cardiomyopathy appears to develop only after long‐term use at high dose, we examined the cardiac effects at 2 or 5 months of meth exposure in female and male mice. We took this opportunity to identify dysregulation of cardiac mRNA that may contribute to, or reflect, cardiac meth toxicity.

Estrogen appears to have a protective effect on the brain of meth‐treated female mice and there are recent clinical observations that men are more susceptible to cardiac damage than women from meth exposure. Therefore, we examined differences in the response of male and female mice to long‐term methamphetamine use by measurements of heart function, cardiac histology, and survival.

## Materials and Methods

### Animal treatments

C57BL/6 mice were 8‐ to 10‐week old at the beginning of this study. Mice were provided standard chow and water ad libitum and housed under a 12‐hr light/dark cycle. They were killed by CO_2_ asphyxiation for tissue extraction and histological analysis after 8 and 20 weeks. Intraperitoneal methamphetamine injections were performed weekly into the left upper quadrant of the abdomen using a 27 g needle. (+) Methamphetamine hydrochloride was purchased from Sigma Aldrich (St. Louis, MO), and dissolved in sterile phosphate‐buffered saline (PBS). The dose was gradually increased 4–5 mg per week for 8 weeks for the 2‐month study, reaching a final dose of 35 mg/kg, or 2 mg per week over 20 weeks to achieve a final dose of 40 mg/kg for the 5‐month study. Control mice were injected with PBS using the same schedule. The 2‐month study was conducted on C57BL/6 female and male mice once, whereas two distinct 5‐month trials were performed on males and one on females. All animal procedures were approved by the Institutional Animal Care and Use Committee at the University of Hawaii.

### Echocardiography

Echocardiograms were performed on unanesthetized mice. The chest area was shaved with an electric razor and depilated with Nair^®^ (Church and Dwight Co, Ewing, NJ). Echocardiograms were performed using the Vevo 2100 high‐resolution imaging system (Visual Sonics, Toronto, Ontario) using a 38 MHz transducer. Mice were acclimated to handling during the days prior to examination. Left ventricular parasternal short‐axis views were obtained from 2D B‐mode and M‐mode images just above the papillary muscles. Diastolic and systolic measurements were taken from M‐mode images containing three consecutive beats. These measurements included left ventricular internal diameter diastole and systole (LVID;s, LVID;d), ejection fraction (EF), and fractional shortening (FS). Data from these measurements were analyzed using VisualSonics software.

### Real‐time PCR

RNA was extracted from homogenized heart tissue using TriReagent^®^ (Molecular Research Center, Cincinnati, OH) following the manufacture's protocol. The RNA was further purified using an RNeasy^®^ Mini Kit (Qiagen, Hildon, Germany), quantified on the Qubit fluorometer (Invitrogen, Carlsbad, CA) and examined on a 1% agarose‐EthBr gel to confirm integrity. Single‐stranded cDNA was synthesized from 1 μg of total RNA using QScript (Quanta Biosciences, Gaithersburg, MD). The mRNA levels of selected dysregulated genes from the microarray results were evaluated by semiquantitative RT‐PCR with Roche Fast Start Universal SYBR Green (Mannheim, Germany) on a 7900HT Fast Real‐time PCR system from Applied Biosystems (Foster City, CA).

### Microarrays

After isolation and purification of RNA, Cy3 labeled nucleotides were incorporated using the Agilent low input quickamp kit (Santa Clara, CA). Individual control and methamphetamine samples from each trial were subjected to first and second strand synthesis followed by in vitro transcription to incorporate the dye into the new strand. After cleanup using the RNeasy^®^ Mini Kit (Qiagen, Hildon, Germany), the samples were hybridized onto Agilent arrays (Mouse GE 4x44K v1) following the manufacturers protocol and scanned using the Agilent G2565CA Scanner. Intensities were extracted and data analyzed using Feature Extraction and Genespring Software (Agilent, Santa Clara, CA).

### Histology

Hearts were perfused by cardiac puncture with PBS followed by 10% formalin in situ. Organs were subsequently fixed in 10% formalin at 4°C overnight and stored in 70% ethanol until embedding. Five‐micron heart sections were deparaffinized and stained with picrosirius red (0.1% sirius red in picric acid). Sections were washed and dehydrated before imaging and analysis. Review and photography were carried out on the Zeiss Axioskop2 photomicroscope with bright‐field illumination and an Olympus CCD color camera interfaced with a Scion CG‐7 frame‐digitizer‐equipped computer. Images were captured using Image J v1.23 acquisition and analysis software (Scion Corporation & NIH).

### Statistics

All results were expressed as mean ± SEM. Statistical significance was evaluated using two‐tailed paired *t*‐test between control‐ and methamphetamine‐treated mice. A value of *P* < 0.05 was considered to be significant for all analyses.

## Results

### Survival

**Figure 1:**
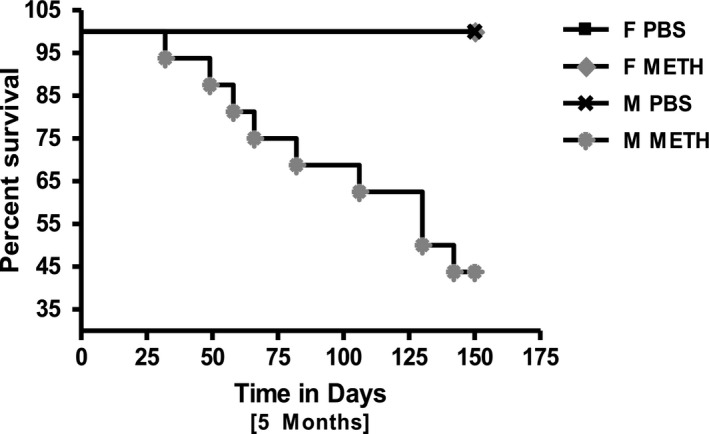
Survival curve of male PBS‐ and Meth‐treated mice (all females survived either treatment). (A) 5‐month PBS (*n* = 4F, *n* = 6M) and Meth (*n* = 7F, *n* = 16M) treated mice. The only premature mortality was seen in Meth‐treated male mice.

C57Bl/6 mice, 8‐ to 10‐week old, were treated weekly with increasing doses of methamphetamine or PBS by I.P. injections for 2 and 5 months. We saw no deaths in control or methamphetamine‐treated animals after 2 months of progressively increasing treatment. In contrast, 5‐month methamphetamine treatment for both sexes (Fig. 1) showed substantial mortality, confined only to males. Fewer than 50% of males survived the 5‐month methamphetamine treatment, whereas no deaths occurred among the females.

### Heart to body weight ratios (HW to BW)

**Figure 2:**
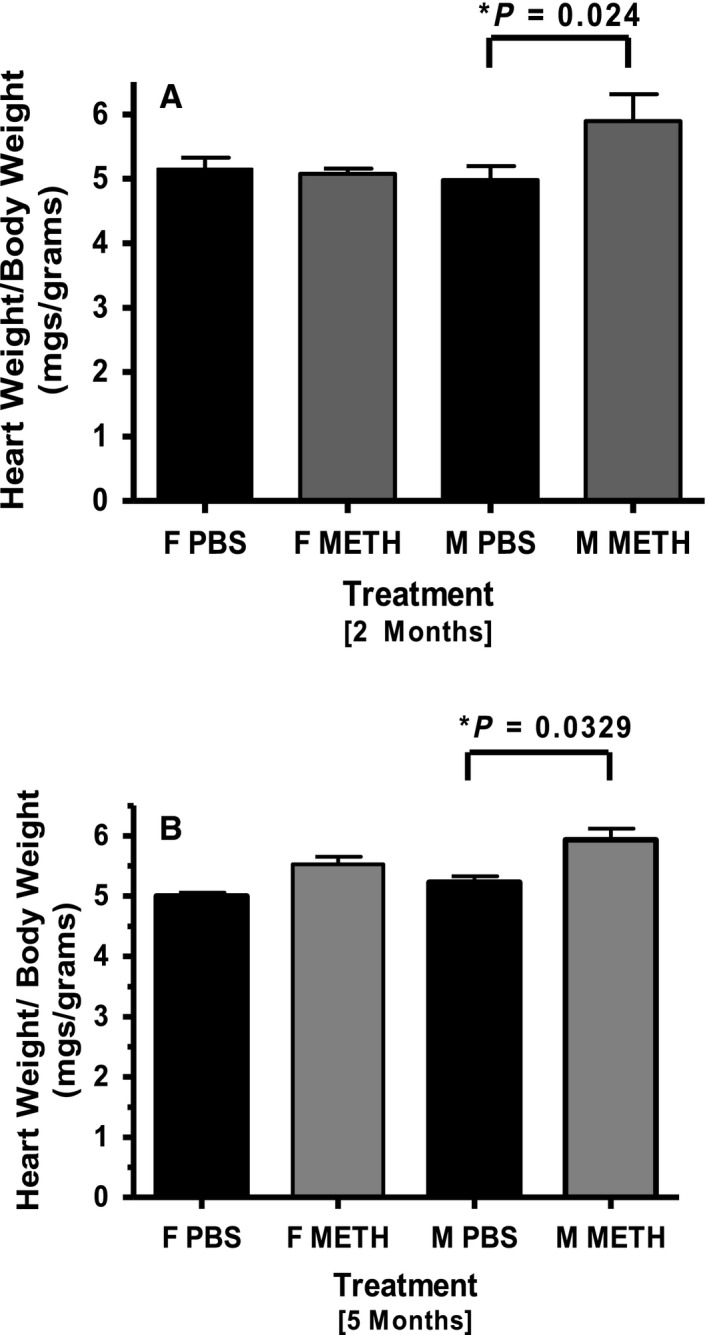
Average HW to BW ratios of PBS‐ and Meth‐treated mice. (A) 2‐month trial showed significant difference between female PBS (*n* = 3) and Meth (*n* = 5) treated mice. Conversely, Meth‐treated males (*n* = 6) had a greater average HW to BW ratio than PBS‐treated controls (*n* = 3). (B) In the 5‐month trial, a small but not statistically significant difference was found between Meth‐treated females (*n* = 7) and PBS controls (*n* = 4). The HW to BW ratio in Meth (*n* = 16) treated males was substantially higher than in males treated with PBS (*n* = 6).

No significant difference between HW to BW ratios in female control (5.16 ± 0.17mgs/g) and methamphetamine (5.08 ± 0.09 mgs/g) treated mice were found in the 2‐month trial. Male‐treated mice (5.89 ± 0.42 mgs/gram) exhibited a much larger change in HW to BW than their controls (4.98 ± 0.22 mgs/grams) (Fig. 2A).

In the 5‐month analysis, HW to BW ratios showed a small increase in treated females (PBS = 5.00 ± 0.60 mgs/gram and meth = 5.53 ± 0.12 mgs/gram) but this did not reach statistical significance. A larger difference that did reach statistical significance was seen in male mice (PBS = 5.23 ± 0.10 mgs/gram and meth = 5.93 ± 0.19 mgs/gram) (Fig. 2B).

Based on the survival rate and echo data from the single 2‐month study and the first 5‐month trial, we felt a larger pool of methamphetamine‐treated male mice was needed to confirm our findings. Hence, a second 5‐month trial was performed only on males, confirming the cardiac hypertrophy in these mice. No significant weight loss was observed for female or male controls, nor for treated animals during the 2‐ and 5‐month study period (data not shown).

### 2‐month echocardiography analyses

Cardiac function was evaluated by unsedated echocardiography three times during each trial. Baseline and 1‐month echo measurements were not significantly different between controls and treated animals. Therefore, only final values are shown in Table 1. At 2 months, there was a trend toward reduced FS and increased LVID in both male and female mice but only the decrease in FS in males reached statistical significance.

**Table 1:** Echocardiographic analyses of 2‐month meth treatment

	Female PBS	Female meth	*P* value	Male PBS	Male meth	*P* value
EF, %	97.2 (±0.36)	95.2 (±1.3)	0.11	97.8 (±0.51)	94.8 (±0.66)	0.12
FS, %	78.04 (±1.8)	69.09 (±2.1)	0.10	79.3 (±1.5)	68.0 (±1.5)	0.01*
LVID;d, mm	2.50 (±0.16)	2.73 (±0.08)	0.48	2.61 (±0.03)	2.75 (±0.10)	0.30
LVID;s, mm	0.55 (±0.03)	0.84 (±0.06)	0.07	0.47 (±0.08)	0.88 (±0.04)	0.05

PBS, phosphate‐buffered saline; EF, ejection fraction; FS, fractional shortening; LVID;d, left ventricular end‐diastolic internal diameter, LVID;s, left ventricular end‐systolic internal diameter. Data are expressed as mean ± SEM and analyzed using a two‐tailed paired *t* test.

### 5‐month female and male echocardiography analyses

**Figure 3:**
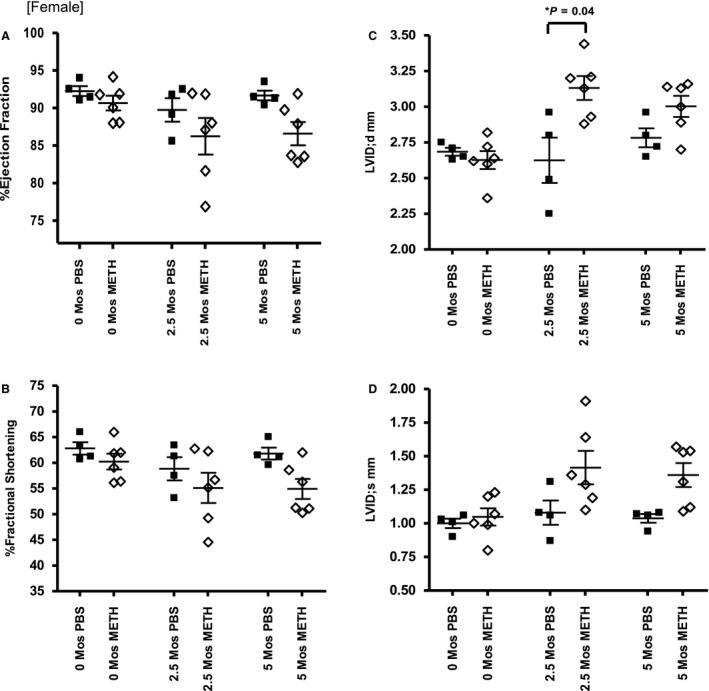
Female 5‐month echocardiography data. (A,B) EF and FS during the 5‐month studies. (C,D) LVID; d and LVID;s measurements at similar time intervals.

Since we saw only modest changes after our 2‐month regimen, and the human pathology is usually seen only after prolonged use, we repeated the study for 5 months with a gradual titration of methamphetamine to a higher final dose. Measurements were taken at baseline, two and a half months and then again at 5 months. Females treated for 5 months exhibited a small but not statistically significant decrement in cardiac function compared to controls (Fig. 3A–D). There was a small decrease in EF and FS between female controls (91.7 ± 0.65%, 61.8 ± 1.2%) and meth‐treated animals (86.6 ± 1.6%, 54.9 ± 2.0%) at 5 months that did not reach statistical significance. The only statistically significant change in ventricular dimension occurred at the two and a half month time point where there was a significant increase between controls (2.6 ± 0.2 mm) and treated females (3.1 ± 0.1 mm) for the end diastolic internal diameter.

**Figure 4:**
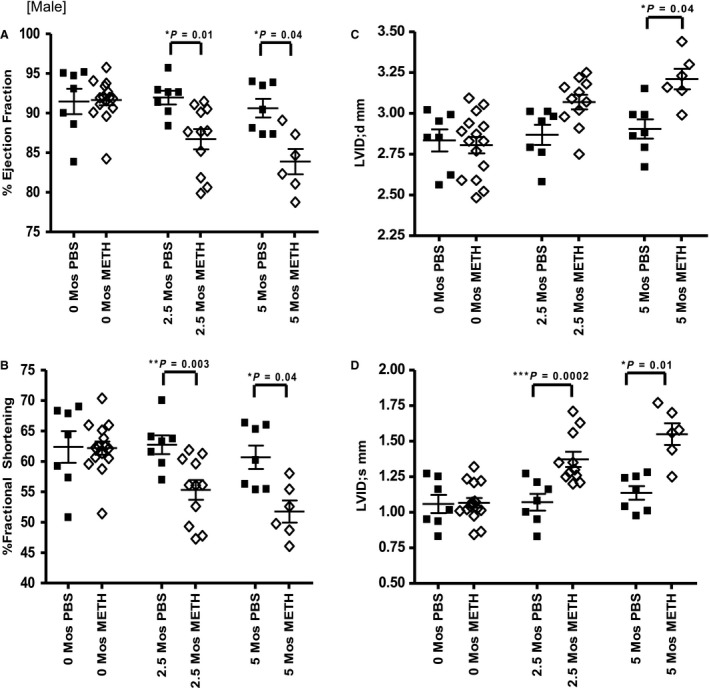
Male 5‐month echocardiography data. (A,B) EF and FS during the 5‐month studies. (C,D) LVID;d and LVID;s measurements at similar time intervals.

In contrast, males had substantial functional and structural changes in the heart (Fig. 4A–D). EF and FS measurements were significantly different between PBS (92.0 ± 0.87, 62.7 ± 1.5%) and meth (86.7 ± 1.3%, 55.3 ± 1.6%) treated mice at two and a half months and more substantial at 5 months (PBS; 90.6 ± 1.2%, 60.7 ± 1.9%, meth; 83.9 ± 1.6%. 51.8 ± 1.8%). LVID;s and LVID;d measurements of PBS (2.9 ± 0.1 mm, 1.1 ± 0.1 mm) and meth (3.2 ± 0.1 mm, 1.6 ± 0.1 mm) treated mice at 5 months dramatically increased confirming the development of dilated cardiomyopathy in the hearts of male mice.

### Histology

**Figure 5:**
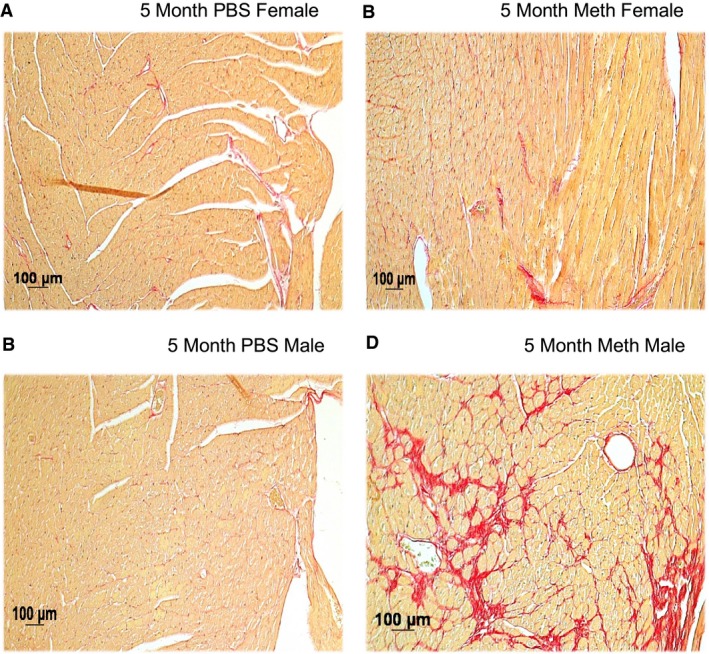
Histology of mouse hearts stained with picrosirius red. Panel B compared to A shows minimal fibrosis in Meth‐treated female mouse heart, whereas the single male mouse that survived 5 months of treatment (panel D) had substantial fibrosis.

Tissue sections of PBS and methamphetamine‐treated hearts are shown in Figure 5A–D. A qualitative measurement was performed to examine collagen deposition in isolated heart sections using picrosirius red. Picrosirius stains collagen fibers red; the intensity and distribution of staining reflects the level of tissue fibrosis. In panels A and B, female 5‐month control and treated heart sections show little to no fibrosis. In panels C and D there is a marked difference in male mice between the methamphetamine‐treated tissue as compared to the control, revealing substantially more fibrosis and damage. No change was observed between the PBS and treated tissue from the 2‐month study (data not shown).

### Microarray data and real‐time confirmation

**Figure 6:**
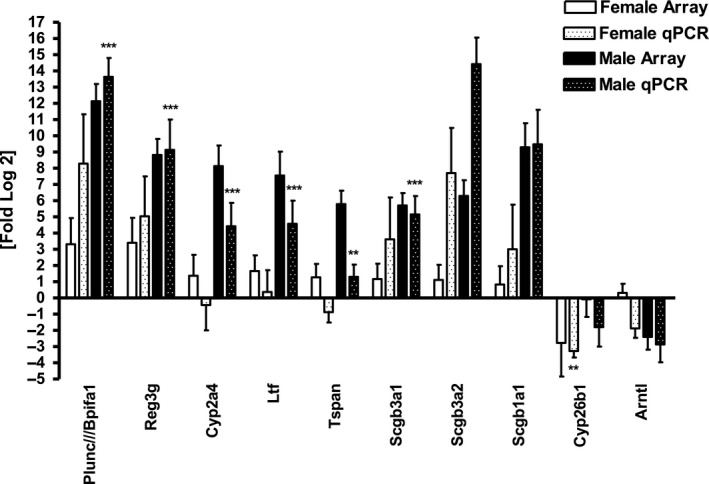
Real‐time confirmation expression patterns of selected highly dysregulated genes from 5‐month chronic Meth‐treated female and male mice. **P < 0.01, ***P < 0.001.

A comparison of array and real‐time expression data from a list of highly dysregulated transcripts after 5 months of meth treatment is shown in Table 2 and graphically in Figure 6. The most highly dysregulated transcripts after meth treatment in female mice were Bpifa1/Plunc, Reg3g and Cyp26b1. The most highly dysregulated transcripts in our male mice were Bpifa1/Plunc, Cyp2a5, Scgb1a1 and Arntl. On our microarrays, Bpifa1/Plunc expression was 3.7‐fold (12.1‐fold, 3.3‐fold) higher in males as compared to females at 5 months. RegIIIg (Reg‐III*γ*) was also more abundant in our females and males showing a 3.4‐fold increase in females and an 8.8‐fold increase in males. Cyp26b1 was downregulated in females by ‐2.77‐fold at 5 months and Arntl was downregulated in males by ‐2.40‐fold for the same time period. Real‐time semiquantitative evaluation confirmed these results.

**Table 2:** Highly dysregulated genes after 5 months of meth exposure

RefSeq	Gene symbol	Fold ∆ by array female (5 month)	Fold ∆ by RT PCR female (5 month)	Fold ∆ by array male (5 month)	Fold ∆ by RT PCR male (5 month)
NM_011126	Bpifa1///Plunc	3.3	8.3	12.1	13.6
NM_011681	Scgb1a1	0.84	3.0	9.3	9.5
NM_011260	Reg3g	3.4	5.0	8.8	9.1
NM_009997	Cyp2a4	1.4	−0.44	8.1	4.4
NM_008522	Ltf	1.7	0.37	7.6	4.6
NM_133681	Tspan1	1.3	−0.88	5.8	1.3
NM_054038	Scgb3a2	1.1	7.7	6.3	14.4
NM_170727	Scgb3a1	1.2	3.6	5.7	5.2
NM_175475	Cyp26b1	−2.8	−3.3	−0.08	−1.8
NM_007489	Arntl	−1.9	0.32	−2.4	−2.9

Highly dysregulated transcripts from 5‐month Meth‐treated mouse model. Only transcripts from known genes that were upregulated by >3.0‐fold or downregulated by <‐2.0‐fold are listed above. Data are presented on a log_2_ scale. *P* value <0.05 for all microarray values. Data represent the average result from three individual microarrays from three separate mice.

All dysregulated transcripts from male mice displayed the same direction of regulation as the microarray data. Transcripts from female mice, which showed lower overall changes in abundance, were confirmed by real‐time PCR only about 70% of the time. Expression levels between females and males listed above were distinct. Males exhibited much higher upregulation than females for Bpifa1/Plunc, Scgb3a2, Scgb3a1 and Reg3g, whereas females showed more downregulation for Cyp26b1 than males. All results have been submitted to GEO under accession # GSE109790.

## Discussion

We describe functional, structural and molecular differences between female and male mice treated with methamphetamine for 5 months. This model demonstrates the cardiac toxicity and dilated cardiomyopathy also seen in human addicts and recapitulates the clinical observation of greater male susceptibility. Very little change was observed in the echocardiography portion of our 2‐month methamphetamine trial in both sexes and in our histology results. Little functional or histological change was observed at 2 months in either sex.

The sexual dimorphism in survival was striking with 56% mortality among males, mostly in the last month of treatment, and none in females. There is an interesting corollary to this finding in human clinical observations where ER visits for chest pain are much higher among male than female meth addicts and decedents show a large male predominance (Karch et al. 1999; Turnipseed et al. 2003; Ito et al. 2009; Herbeck et al. 2015).

We performed an in vivo assessment of the heart using echocardiography and observed a decrease in cardiac function in males after 5 months of progressively increasing methamphetamine injections. In our 2‐month study, our results reflect an earlier stage in the myopathic process. Although there was a decrease in fractional shortening between treated and control males, as well as a significant increase in the average male HW to BW ratio, we did not detect any mortality or myocardial fibrosis in males or females.

In our 5‐month study, in addition to the marked mortality, we saw a substantial increase in HW to BW ratio, significant decrement in cardiac function and a remarkable increase in the amount of cardiac fibrosis in male compared to female mice. It is evident that dilated cardiomyopathy develops more slowly in methamphetamine‐treated female mice than males. Although not amenable to statistical evaluation, the fibrosis seen in the single surviving male illustrates another important detrimental effect of methamphetamine exposure, which likely contributes to the functional and gravimetric effects that we observed. It is possible that we could be under‐estimating the effect of meth on cardiac function in the male mice, as the mice that died may have had worse function than those that lived, (although we cannot prove this).

To further evaluate the underlying molecular basis for the changes seen in our model we performed transcriptional analysis on the hearts of treated and control mice. We identified several dysregulated transcripts involved in inflammation, metabolic processes and circadian rhythm. Bpifa/Plunc was the most dysregulated transcript. It is thought to have antimicrobial as well as anti‐inflammatory functions in the trachea but is not commonly encountered in the heart, although we have previously found it to be upregulated in a model of chronic doxorubicin toxicity and downregulated in a cardiomyocyte‐specific endothelin‐1 knockout (Yi et al. 2006; Bingle and Bingle 2011; Gally et al. 2011; Lukinskiene et al. 2011).

Other transcripts related to inflammation were also more abundant in methamphetamine exposed hearts. RegIIIg, Scgb3a2 and IL6 were substantially upregulated in both females and males with the exception of IL6 which was only slightly upregulated in meth‐treated males. IL6, a cytokine produced by inflammatory cells, is responsible for regulation of cell growth, apoptosis, differentiation, and survival in various cell types and organs (Banerjee et al. 2009; Rincon and Irvin 2012). It has pleotropic effects in the heart but is generally thought to be profibrotic (Banerjee et al. 2009). It is thus unclear why IL6 transcript was more abundant in our female mice, however, the function of this cytokine depends on the abundance of its receptor and other aspects of the local milieu, which could be modified by estrogen.

The transcripts for RegIIIg and Scgb3a2 were more abundant in our treated male mice than our females. RegIIIg is known to play a role in innate immune functions and bacterial colonization of the gut (Wu and Chakravarti 2007; Johansson and Hansson 2011; Matsumoto et al. 2012). Like RegIIIg, Scgb3a2 is predominantly expressed in the pulmonary airway epithelium and plays a role in lung inflammation (Chiba et al. 2006; Lu et al. 2011). The marked upregulation of Bpifa/Plunc, RegIIIg and Scgb3a2 suggests a surprising role for immune mediators not previously appreciated to have important roles in methamphetamine cardiomyopathy.

In summary, we have developed a chronic mouse model of meth‐induced cardiomyopathy that is similar to the clinical presentation seen in human patients. We have determined that long‐term meth exposure increases HW to BW ratios in both male and female mouse hearts while substantially reducing the survival rate of our males. It appears that this long‐term exposure is important to obtain and evaluate cardiac pathology, especially as these changes were not seen with a shorter, 2‐month, treatment schedule.

Additionally, with the long‐term protocol that we used, meth treatment contributes to the development of cardiomyopathy in male mice with less effect on females. We have identified several highly dysregulated transcripts, Bpifa/Plunc, IL6, RegIIIg, and Cyp26b1, that may play a role in heart protection or injury and may do this by regulating important pathways involved in inflammation or toxicity. It is interesting that we find a gender discordance in rates of death, HW to BW ratios, cardiac dilation, and histological evidence of fibrosis. Although the hearts of treated female mice do show some evidence of dilation and dysfunction, death, perhaps due to dysrhythmia, is disproportionately found in the males. One possible explanation could be that estrogen has a greater protective role in the heart during the course of drug use than previously thought. Another possibility is that females metabolize methamphetamine more efficiently and/or with less toxicity and that this pharmacokinetic effect protects the female heart. It will be important to determine how estrogen, or female gender, affects meth metabolism in long‐term users. Future studies in rodents may help to elucidate how this popular and devastating drug selectively damages the heart in men.

## Conflict of Interest

None declared.

## Supporting information




**Table S1.** RNA abundance in the heart after 5 months of meth treatment. All transcripts > 3‐fold more abundant or < 2‐fold less abundant are shown for both male and female mice.
