# Supplementary material for: Sex influences susceptibility to methamphetamine cardiomyopathy in mice
Source: Physiol Rep. 2019 Mar 19;7(6):e14036. doi: 10.14814/phy2.14036 (PMC6424857; doi:10.14814/phy2.14036)
Supplement: Supplementary file 1 [file PHY2-7-e14036-s001.docx]

Supplemental Table I

GeneName (or ID)	GeneSymbol	RefSeqAccession	Female		Male	
			Fold Change	P-value	Fold Change	P-value
BPI fold containing family A, member 1///palate, lung, and nasal epithelium associated	Bpifa1///Plunc	NM_011126	3.319	0.004	12.129	<0.001
cytochrome P450, family 2, subfamily a, polypeptide 5	Cyp2a5	NM_007812	1.122	0.381	9.655	<0.001
secretoglobin, family 1A, member 1 (uteroglobin)	Scgb1a1	NM_011681	0.835	0.067	9.293	<0.001
regenerating islet-derived 3 gamma	Reg3g	NM_011260	3.406	0.012	8.824	<0.001
cytochrome P450, family 2, subfamily a, polypeptide 4	Cyp2a4	NM_009997	1.189	0.21	8.786	<0.001
cytochrome P450, family 2, subfamily a, polypeptide 4	Cyp2a4	NM_009997	1.146	0.411	8.775	<0.001
lactotransferrin	Ltf	NM_008522	1.613	0.039	8.252	<0.001
cytochrome P450, family 2, subfamily a, polypeptide 22	Cyp2a22	NM_001101467	0.79	0.62	7.199	<0.001
lactotransferrin	Ltf	NM_008522	1.719	0.075	6.804	<0.001
SEC14-like 3 (S. cerevisiae)	Sec14l3	NM_001029937	1.763	0.08	6.782	<0.001
cytochrome P450, family 2, subfamily a, polypeptide 4	Cyp2a4	NM_009997	1.763	0.08	6.782	<0.001
tetraspanin 1	Tspan1	NM_133681	1.025	0.959	6.327	<0.001
secretoglobin, family 3A, member 2	Scgb3a2	NM_054038	1.111	0.252	6.308	<0.001
secretoglobin, family 3A, member 1	Scgb3a1	NM_170727	1.172	0.097	5.699	<0.001
A_52_P61752			1.52	0.227	5.241	<0.001
keratin 15	Krt15	NM_008469	0.935	0.87	5.125	<0.001
cytochrome P450, family 2, subfamily f, polypeptide 2	Cyp2f2	NM_007817	1.071	0.332	5.019	<0.001
BPI fold containing family B, member 1///cDNA sequence U46068	Bpifb1///U46068	NM_001012392	1.386	0.109	4.985	<0.001
transmembrane protein 212	Tmem212	NM_001164437	1.95	0.036	4.759	0.002
trefoil factor 2 (spasmolytic protein 1)	Tff2	NM_009363	1.22	0.506	4.617	<0.001
A_52_P352735			0.97	0.867	4.381	0.001
SEC14-like 3 (S. cerevisiae)	Sec14l3	NM_001029937	0.949	0.861	4.148	<0.001
aquaporin 5	Aqp5	NM_009701	0.847	0.268	4.006	<0.001
family with sequence similarity 47, member E	Fam47e	NM_001033478	1.411	0.185	3.963	0.001
dynein light chain roadblock-type 2	Dynlrb2	NM_029297	0.984	0.832	3.901	<0.001
surfactant associated protein D	Sftpd	NM_009160	0.574	0.027	3.825	0.001
A_52_P656455			0.793	0.284	3.818	<0.001
dynein light chain roadblock-type 2	Dynlrb2	NM_029297	1.082	0.598	3.788	<0.001
chemokine (C-X-C motif) ligand 17	Cxcl17	NM_153576	0.935	0.833	3.777	<0.001
leucine rich repeat containing 26	Lrrc26	NM_146117	1.083	0.795	3.718	0.001
A_52_P502849			1.236	0.333	3.705	<0.001
RIKEN cDNA 5330417C22 gene	5330417C22Rik	NM_001033304	0.796	0.342	3.651	0.001
immunoglobulin lambda variable 1	Iglv1		1.12	0.732	3.619	0.001
sulfotransferase family 1D, member 1	Sult1d1	NM_016771	1.024	0.936	3.6	0.001
cDNA sequence BC048546	BC048546	NM_001001179	1.181	0.701	3.568	<0.001
A_52_P391639			0.783	0.365	3.551	<0.001
anterior gradient 2 (Xenopus laevis)	Agr2	NM_011783	1.841	0.019	3.525	<0.001
forkhead box J1	Foxj1	NM_008240	1.12	0.746	3.484	<0.001
WAP four-disulfide core domain 2	Wfdc2	NM_026323	0.94	0.497	3.472	0.001
A_51_P203148			1.574	0.101	3.375	0.003
EF-hand calcium binding domain 10	Efcab10	NM_029152	1.414	0.627	3.314	<0.001
mucin 5, subtype B, tracheobronchial	Muc5b	NM_028801	1.207	0.636	3.303	0.001
predicted gene 129	Gm129	NM_001033302	1.049	0.822	3.234	0.002
adenylate kinase 7	Ak7	NM_030187	1.033	0.929	3.2	0.001
A_51_P461902			1.271	0.159	3.193	0.003
RIKEN cDNA 1600029D14 gene	1600029D14Rik	NR_028123	1.322	0.407	3.182	0.012
A_52_P859747			1.02	0.924	3.182	<0.001
tetraspanin 1	Tspan1	NM_133681	1.447	0	3.179	0.011
A_51_P407657			2.236	0.014	3.147	0.007
A_52_P337690			1.898	0.049	3.124	0.002
A_52_P512769			1.121	0.673	3.099	0.004
immunoglobulin joining chain	Igj	NM_152839	1.253	0.114	3.087	0.002
keratin 23	Krt23	NM_033373	1.332	0.315	3.076	0.003
immunoglobulin heavy chain (J558 family)	Igh-VJ558		1.432	0.068	3.059	0.002
A_52_P383114			1.316	0.41	3.053	0.029
A_52_P614207			1.174	0.392	3.048	<0.001
A_52_P149235			1.161	0.421	3.035	0.001
A_52_P600555			2.371	0.088	3.034	<0.001
